# The Growth and Scope of Voluntary Hospitals in London

**Published:** 1897-08-14

**Authors:** 


### The Institutional Workshop.

### THE GROWTH AND SCOPE OF VOLUNTARY HOSPITALS IN LONDON.

From a Correspondent.

### IV.—THE EXTENSION OF LYING-IN CHARITIES.

Not many years passed from the foundation of the first lying-in hospital before a certain reluctance to accept the conditions and benefits offered became apparent on the part of many objects of these charities. Anxious mothers dreaded the effect of their absence on home and children; the less civilised feared the loss of liberty involved; and the timid shrank from entering a hospital for their confinement as something new, and, therefore, to be dreaded. It was found, moreover, to be an expensive matter. The women often came in weeks before it was necessary, and required very nourishing food during the whole period. It is true that in some cases a charge for board was made when the patients extended their stay beyond three weeks or a month, but the charge can hardly have been remunerative, and must often have remained unpaid. Weighing all these circumstances, it became evident that, to afford really efficient aid to the mothers of London, some other plan must be adopted.

Accordingly, in 1757, a society was founded under the name of "The Lying-in Charity for Delivering Poor Married Women at their own Habitations"—the same which is now known as the Royal Maternity Society. Several new features were inaugurated by the promoters of this charity. Out-patients had long been received at the general hospitals, but house visitation had formed no part of the plan of any institution up to this time. The new charity provided that each patient should be visited in her own home before, at, and after delivery, and until she was recovered, and the physicians and midwives in this way gained an entrance into regions never before visited except by the officers of justice or, more rarely, by the priest. The procedure and medical etiquette in matters obstetrical differed widely from that observed in the present day. The delivery of the mother was performed by practitioners known as men-midwives, who reserved themselves solely for this branch of their profession. They acted, in the case of wealthy patients, under the supervision of a physician, whose part consisted in the medical care of the patient before and after delivery. Surgeons had no part in this branch of practice, and, indeed, the constitutions of some of the medical charities of the eighteenth century expressly exclude the appointment of any to the office of house-surgeon who practise midwifery and pharmacy. Among the middle and lower classes the employment of female midwives was almost universal; some, no doubt, had acquired by constant practice a high degree of skill, but any old woman who chose could call herself a midwife, and the rule was ignorance and drunkenness. The old impressive ceremony by which an oath was administered to midwives before they were allowed to practise, and solemn authority bestowed on them to baptise infants in case of imminent danger, had fallen after the Reformation into disuse, and Government has been slow to recognise the need for replacing it by regulations more suited to the times. But the training of women in midwifery advanced rapidly under the new hospitals, and was urged upon the public as not the least of the benefits they procured for the poor by their subscriptions. The new Lying-in Charity soon had a trained service of 20 or 30 midwives at their disposition. "On account of future benefit to themselves" they engaged to serve the charity for two years at a low price, and were then accustomed to set up on their own account among those who could afford to pay for their services.

Another new feature in this charity was the limitation of the area within which the relief was given. It must be confessed that the boundaries were sufficiently comprehensive, embracing in fact the whole area of London as it then existed. They were: Westminster Bridge, Millbank, Pimlico, Hyde Park Corner, and Tyburn (now the Marble Arch) turnpikes; Portman Square, Marylebone, Tottenham Court, Gray's Inn Lane end, St. John Street, Pentonville (as far as the chapel), and Islington, to Cross Street, inclusive; Goswell Street turnpike, the City Road, and Shoreditch Workhouse, Hackney turnpike, Bethnal Green turnpike, near the church, and to the Grove House, Mile End Old Town, Limehouse Hole, Rotherhithe Church, Grange Road, and Kent Street end, Blackman Street turnpike, and the road from Blackman Street to Westminster Bridge. The object of the limitation was, no doubt, to protect the midwives from the waste of time which would be incurred in journeys to applicants residing in the remote villages, which are now populous suburbs.

The success of this new method of relieving the poor was so well marked that the Westminster Lying-in Hospital adopted the same plan as an adjunct to the work of the lying-in wards. They do not appear to have imposed any limit of distance, but the midwives all resided within the Westminster district, and each received the applications of the persons living nearest to them.

In 1780 another lying-in society, under the name of the "Benevolent Institution for the Sole Purpose of Delivering Poor Married Women at their own Habitations," was founded. The same methods were adopted as in the former society, and very much the same area was defined for the operations of the charity, except that in this case the district on the other side of the river, including Lambeth, was taken in, as well as the villages of Chelsea, Brompton, Knightsbridge, and Kensington.

It may well be believed that many of the homes into which the physicians and midwives penetrated in the service of these charities were wanting, not merely in every comfort, but in the barest necessaries for the use of the patients. In this particular they were no better—they could hardly have been worse—than the homes visited in the present day on the same errand. The element of over-crowding, which is so painful a feature of to-day, had hardly yet become so strongly marked, except, perhaps, in one or two of the worst localities. But if the numbers were less, the atmosphere was far more polluted. The iniquitous window-tax had deprived the poor of their best inheritance of sun and air; sanitary regulations, constantly enacted, were as constantly defied, and some of the poorer districts of the town remained until well into the middle of this century without water and without drainage. The ruinous condition of the hovels of the poor was, perhaps, the best protection of the inhabitants, for the gaping roofs and ill-fitting doors at least gave entrance to the air, though too often that was foully contaminated by the garbage of the narrow streets and alleys. It does not appear that it was part of the plan of any of these lying-in charities at the beginning to furnish linen or food to the poor who were attended in their own homes. The credit of organising this additional benefit must be given to Mrs. Wakefield, who, in 1791, founded a lying-in society in the parish of Tottenham High Cross, north of London, under a new system of small weekly contributions from ladies interested in the poor of the neighbourhood. By an entrance fee of 3s., and the payment of 6d. a week, each subscriber was enabled to recommend an "object" to the benefits of the society, which included the attendance of nurse and midwife, and the loan of a bag of linen. The outlay was so moderate, and the benefits conferred so gratefully acknowledged, that the scheme increased with astonishing rapidity, and was soon no longer limited to lying-in patients. In varoius parts of the country kindred charities were formed on Mrs. Wakefield's model, and her bag of linen now forms an essential part of every lying-in society.

The chain of relief, to the mothers of the metropolis, soon supplemented in many districts by further efforts, might now be considered fairly complete. All classes were benefited by the stimulus given to the science and art of midwifery in the observation of thousands of cases yearly, which were formerly hidden in the obscure practice of ignorant, and often mischievous, women. The middle classes could be attended at moderate cost by midwives trained under the direct observation of the best doctors of the day. The poor met with ready relief and attention, either in hospitals situated conveniently for their advice and reception, or in their own homes, to which some dim echo of comfort and cleanliness may have penetrated in the wake of the skilled attendance, and of the ladies who made it their business to visit cases of this description. The homeless vagrant poor, whose history in every nation and period has remained unwritten, might receive in their last extremity a refuge in the workhouse. There food, drink, and shelter were accorded them, and the rude offices of the practitioner appointed to such cases. There, too, they might receive their finishing touch in the education of vice and immorality. To rescue the honest poor in sickness and adversity from the associations and degradation of the workhouse was one of the strongest spurs to liberality among the benevolent of this period.

